# Fine-scale genetic structure among greater sage-grouse leks in central Nevada

**DOI:** 10.1186/s12862-016-0702-4

**Published:** 2016-06-14

**Authors:** Joshua P. Jahner, Daniel Gibson, Chava L. Weitzman, Erik J. Blomberg, James S. Sedinger, Thomas L. Parchman

**Affiliations:** Program in Ecology, Evolution, and Conservation Biology, University of Nevada, Reno, NV 89557 USA; Department of Biology, University of Nevada, Reno, NV 89557 USA; Department of Natural Resources and Environmental Science, University of Nevada, Reno, NV 89557 USA; Department of Wildlife, Fisheries, and Conservation Biology, University of Maine, Orono, ME 04469 USA

**Keywords:** *Centrocercus urophasianus*, Genotyping-by-sequencing, Lek mating, Population genomics, Population structure, Reproductive skew

## Abstract

**Background:**

Mating systems that reduce dispersal and lead to non-random mating might increase the potential for genetic structure to arise at fine geographic scales. Greater sage-grouse (*Centrocercus urophasianus*) have a lek-based mating system and exhibit high site fidelity and skewed mating ratios. We quantified population structure by analyzing variation at 27,866 single-nucleotide polymorphisms in 140 males from ten leks (within five lek complexes) occurring in a small geographic region in central Nevada.

**Results:**

Lek complexes, and to a lesser extent individual leks, formed statistically identifiable clusters in ordination analyses, providing evidence for fine-scale geographic genetic differentiation. Lek geography predicted genetic differentiation even at a small geographic scale, which could be sharpened by strong site fidelity. Relatedness was also higher among individuals within lek complexes (and leks), suggesting that reproductive skew, where few males participate in most of the successful matings, could also potentially contribute to genetic differentiation. Models incorporating a habitat resistance surface as a proxy for potentially reduced movement due to landscape features indicated that both geographic distance and habitat suitability (i.e. preferred habitat) predicted genetic structure, with no significant effect of man-made barriers to movement (i.e. power lines and roads). Finally, we illustrate how data sets containing fewer loci (<4000) had less statistical precision and failed to detect the full degree of genetic structure.

**Conclusion:**

Our results suggest that habitat features and lek site geography of sage-grouse shape fine scale genetic structure, and highlight how larger data sets can have increased precision and accuracy for quantifying ecologically relevant genetic structure over small geographic scales.

**Electronic supplementary material:**

The online version of this article (doi:10.1186/s12862-016-0702-4) contains supplementary material, which is available to authorized users.

## Background

Population genetic studies are essential for understanding how behavioral, ecological, and evolutionary processes shape the geographic distribution of genetic variation. Recent DNA sequencing advances now allow variation to be readily assayed at thousands of loci for large numbers of individuals and populations in non-model organisms [1, 2], potentially illuminating the relationships among ecological, genetic, and geographic variation at previously unattainable scales [3–6]. These approaches have value for uncovering previously unrecognized patterns of genetic structure, generating more precise estimates of demographic and evolutionary parameters, and providing increased opportunity for understanding adaptation [7–10]. Such approaches also have special promise for analyses of genetic variation related to contemporary ecological processes and population demography in species of ecological or conservation significance [7, 11, 12].

Variation in behavioral and life history traits may have important consequences for the geographic structuring of genetic variation across populations (e.g. [13–16]). For example, a number of avian species have lek-based mating systems in which a small number of males contribute disproportionately to reproduction, dispersal is restricted by strong site fidelity, and leks (i.e. mating arenas) remain at the same geographic locations for long periods of time [17–21]. Strong site fidelity could reduce gene flow among leks and give rise to spatial genetic structure even in the absence of geographic barriers. Because a subset of males typically contribute disproportionately to reproduction on leks, reproductive skew (i.e. where few males participate in most of the successful matings) can also lower effective population size (*N*_e_) and increase genetic drift [17, 22, 23]. These factors, in addition to the potential for kin selection in lek-based mating systems [24, 25], could enhance the effects of drift in structured populations, give rise to fine-scale spatial genetic structure, and increase extinction probability of isolated populations [17, 26–29].

Greater sage-grouse (*Centrocercus urophasianus*; hereafter sage-grouse) are an iconic western North American bird species with a lek-based mating system [30]. Male sage-grouse display for females at leks residing in open patches of sagebrush (*Artemesia* spp.), where a subset of dominant males can account for a large proportion of the successful matings ([31]; but see [32]). Degradation of sagebrush ecosystems across North America has substantially reduced the geographic range and abundance of sage-grouse over the last century. Population declines ranged from 45 to 90 % across the distribution and local extirpations have occurred in many regions [33, 34]. Habitat degradation and population declines [35, 36] have raised concerns about population persistence (but see [37] for an alternative viewpoint), though sage-grouse were recently found unwarranted for listing under the U.S. Endangered Species Act [38]. Low movement rates among leks due to high site fidelity (e.g. [39]), in addition to reproductive skew, could interact with habitat variation to limit gene flow, enhance genetic drift, and shape fine-scale genetic structure in sage-grouse populations, as has been found in several lek-breeding species (e.g. [17, 20, 40]).

Previous studies utilizing small data sets of traditional molecular markers (e.g. mtDNA; microsatellites) have reported variable patterns of genetic structure across sage-grouse populations, leaving uncertainty about the scale of geographic genetic structure and obscuring the expected link between demographic and population genetic parameters across the range [41]. The detection of genetic differentiation across broad geographic scales resulted in the recognition of three distinct populations: Gunnison sage-grouse (*C. minimus*), greater sage-grouse, and the Bi-state population of greater sage-grouse (spanning the California and Nevada border) [42–44]. However, most studies at finer geographic scales have not identified genetic structure among geographically proximate leks [45, 46], leaving uncertainty about the potential for site fidelity and reproductive skew to influence genetic structure [45, 47, 48]. In contrast, a recent study of geographically proximate leks in northwestern Colorado documented among-lek differentiation and evidence for isolation by distance [49]. Although these studies suggest movements among leks may limit differentiation in some parts of the range, higher resolution data sets should improve our understanding of the geographic scale of genetic structure and the factors associated with it.

**Fig. 1:**
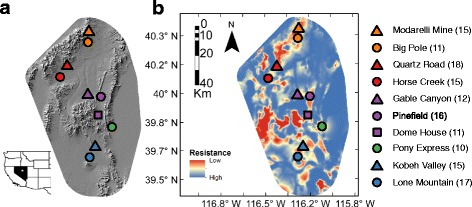
Locations of leks within Eureka County, NV, USA. The study area varies across the landscape in both (**a**) topology and (**b**) habitat resistance. The habitat resistance surface was derived from the inverse of a sage-grouse nest selection model [73] and included the following variables (and direction of effect): 1) nest site elevation (−); 2) slope (+); 3) slope * elevation (−); 4) amount of habitat classified as sagebrush with 1000 m (+); 5) distance from nearest lek (−); 6) amount of habitat classified as sagebrush * distance to lek (−); 7) amount of habitat classified as pinyon-juniper woodlands (+); 8) amount of habitat classified as pinyon-juniper woodlands^2^ (−); 9) distance from nearest water source (−); and 10) distance from nearest water source^2^ (−). Warm colors are indicative of areas dominated by sagebrush or other shrubs, while cool colors are indicative of playas, pinyon-juniper woodlands, exotic grasslands, and pivot agricultural fields. The legend is ordered from highest to lowest latitude, with the number of individual sage-grouse sampled at each lek listed parenthetically. Leks within the same lek complex share the same color (North Cortez = *orange*; Cortez = *red*; Pine Valley = *purple*; Pony Express = *Green*; Kobeh Valley = *blue*)

Here we use a genotyping-by-sequencing (GBS) approach to quantify genetic variation at 27,866 single-nucleotide polymorphisms (SNPs) across 140 sage-grouse from ten leks located throughout a small geographic region in central Nevada (Fig. 1). Very strong breeding-site fidelity has been documented for these specific leks across multiple years of monitoring, where 330 of 345 (98.5 %) among-year observations involved males returning to the same leks [39]. We focus on five groups of leks with low rates of inter-group movement (i.e. lek complexes) to quantify genetic structure among leks and lek complexes and the extent to which geography and habitat suitability predict genetic differentiation. Our results indicate that geography, habitat, and potentially behaviors associated with a lek-based mating system may contribute to genetic differentiation and highlight the utility of large SNP data sets for characterizing genetic structure at fine geographic scales.

## Methods

### Sage-grouse sample collection

During the spring breeding season, we sampled ten leks separated by an average distance of 35.7 km in Eureka County, Nevada (Fig. 1; Additional file 1: Table S1) with known histories of individual movements and sufficiently large sample sizes [39]. Although 4–5 additional leks occur within this immediate area, the sampled leks have been the subject of past behavioral and ecological studies, remained constantly active from 2003 to 2012, and were evenly distributed across the region [39]. *A priori*, we grouped leks into five lek complexes based on geographic proximity (<15 km), patterns of male movements documented across multiple years of monitoring, and potential habitat barriers [39]. Observed movements among leks were highly uncommon (only 1.5 % of 355 males moved between leks), but typically occurred within the same lek complex [39]. Two state highways, unpaved secondary roads, and power transmission lines are present in the study area, but sage-grouse regularly crossed these infrastructures (based on 10 years of radio-telemetry data; DG, EJB, and JSS, unpublished data). There are no major geographic barriers to dispersal in the study area, but it is possible that individuals are less likely to move through low-quality habitat. Sage-grouse here make regular seasonal movements from spring breeding leks to higher elevation summer/fall habitat (>2100 m): individuals from the Pine Valley, Kobeh Valley, and Pony Express lek complexes utilize the Roberts Creek Range, while individuals from the Cortez and North Cortez lek complexes typically occupy the Cortez Range [50, 51]. Although birds from different leks and lek complexes share summer habitat and can have large yearly and summer movement distances (Additional file 1: Figure S1), nearly all birds return to their specific lekking grounds each spring [39]. Thus, the sage-grouse in our study area exhibit strong lek-site fidelity.

We captured 140 male sage-grouse on these leks during the spring over a 5 year period (March–May, 2007–2012). We only included males in our study because we were unable to collect a sufficient number of female samples. Approximately 1.5 ml of blood was drawn from the basilic vein of each captured bird. Protocols for the capture and handling of sage-grouse were approved by the University of Nevada, Reno Institutional Animal Care and Use Committee (Protocol Numbers A02/03-22, A05/06-22, A07/08-22, A09/10-22).

### Genotyping-by-sequencing library preparation

DNA was extracted from blood using Qiagen DNeasy Blood and Tissue kits (Qiagen Inc., Valencia, CA) and quantified using a Nanodrop spectrophotometer (Thermo Scientific Inc., Waltham, MA). Reduced-representation libraries were constructed using a GBS approach [52, 53]. Genomic DNA was first cut with two restriction enzymes, EcoRI and MseI, and a unique DNA barcode was ligated to digested fragments from each individual. Each EcoRI adaptor consisted of bases matching the endonuclease cut site, a unique eight to ten base pair DNA barcode sequence, and an Illumina adaptor; the MseI adaptors consisted of the endonuclease cut site and the opposite Illumina adaptor. Uniquely barcoded ligation products from all individuals were pooled and PCR-amplified using standard Illumina primers. Libraries were size-selected for a region between 350 and 450 bases using a BluePippin quantitative electrophoresis unit (Sage Science, Beverly, MA). We generated single end, 100 bp reads on one lane of an Illumina HiSeq 2500 platform at the University of Texas Genomic Sequencing and Analysis Facility (Austin, TX).

### Assembly and variant calling

Illumina reads were first filtered to remove potential contaminant DNA (PhiX, *E. coli*) and low quality or aberrant reads. We used a Perl script to recognize barcodes assigned to each individual bird, to correct single-base errors in barcode sequences, and to remove sequences containing portions of the Illumina adaptors. After removing barcodes and cut site bases, retained sequences were 87, 88, or 89 bp in length. A reference of GBS sequenced regions was created with *de novo* assembly of a random subset of 25 million reads using the SeqMan ngen software (DNASTAR, Inc.). This used a minimum match percentage of 95, a gap penalty of 30, and only retained contigs containing a minimum of 10 reads (additional assembly parameters are available from the authors by request). We generated the reference by removing contigs that were over-assembled or that were shorter than 84 or longer than 90 bases. Reads from each bird were subsequently aligned to the reference using the aln and samse algorithms in bwa (Burrows-Wheeler Aligner; [54]).

The number of reads representing alternative nucleotide states (i.e. SNPs) at each variant site was quantified using samtools and bcftools [55]. We only called SNPs when 90 % of the individuals had at least one read at the locus. Genotype likelihoods were calculated with bcftools, stored in Variant Call Format, and converted to a composite genotype likelihood format for downstream analysis. We excluded variable sites with more than one alternate allele and loci with minor allele frequencies less than 5 %. For contigs containing more than one SNP, we randomly selected a single SNP to increase the independence of loci used in subsequent analyses. Assembly related files and genotype matrices are available at Dryad (doi:10.5061/dryad.652r5), and additional parameter settings for these analyses are available from the authors by request.

We used a hierarchical Bayesian model that incorporates uncertainty in sequencing coverage across loci and individuals to estimate allele frequencies and genotype probabilities simultaneously for all individuals based on estimated genotype likelihoods [52]. This model treats allele frequencies as priors, and coincidentally estimates allele frequencies and genotype probabilities while incorporating uncertainty arising from variation in sequence coverage. Thus, the estimates incorporate information on the probability of sampling each of the two alleles at a locus from the population, as well as coverage. We obtained posterior estimates of genotype probabilities by running 10,000 MCMC steps after a 6000 step burn-in and thinning every other step. These genotype probabilities were then converted to composite genotype values, where an individual’s genotype ranged from 0 to 2 at a locus (values of 0 and 2 represent homozygotes for different alleles, while values of 1 represent heterozygotes).

### The relationship between geography and genetic structure

To summarize genotypic variation across individuals and to explore the relationship between geography and genetic structure, we utilized three complementary ordination methods [56]. For all three approaches, we implemented permutational multivariate analysis of variance (PERMANOVA; [57]) in the vegan package [58] in R [59] as a *post hoc* test of differentiation among leks and lek complexes based on Euclidean distances of the first two ordination axes.

We first conducted principal component analysis (PCA) on genotype probabilities using the *prcomp* function in R. To quantify the relationship between individual genotypic variation and geography, we conducted Procrustes analyses on principal components (PCs) [60, 61] using the vegan package in R. We rotated a genomic matrix of PC1 and PC2 onto a geographic matrix of latitudes and longitudes and used 999 permutations to test significance of Procrustes correlations. To test the hypothesis that sage-grouse within a lek complex are more genetically similar to one another than expected, we calculated the Euclidean genetic distance among all individuals using the first two PCs. For each subset, the observed genetic distance of individual pairs sharing the same lek complex was compared to a null distribution of genetic distances constructed from 1000 permutations of lek complex-sharing status, which was randomly assigned among pairs. The same analyses were conducted at the lek level.

We conducted Discriminant Analysis of PCs (DAPC) [62] using the adegenet package [63, 64] in R. Unlike PCA, DAPC maximizes the ratio between the variance explained between groups and the total variance explained [56] and is often used to group individuals into clusters. DAPC is useful for GBS datasets because the analysis effectively reduces the dimensionality of large datasets while delineating population genetic structure in a somewhat analogous manner to more computationally intensive Bayesian clustering methods (e.g. STRUCTURE; [65]) [62]. To reduce over- or under-discrimination, we selected the number of retained PCs by predicting the maximum α-score with the *optim.a.score* function (20 replicate α-scores were calculated), following [62]. We first conducted DAPC without *a priori* group assignments by inferring the most likely number of genetic clusters (K) using the *find.clusters* function in the adegenet package. This function utilizes K-means clustering to calculate a Bayesian information criterion (BIC) value for each potential value of K (the most likely K has the lowest BIC value) and delineates individual group assignments for DAPC [62]. Additionally, we conducted DAPC using *a priori* group assignments at both the lek (*K* = 10) and lek complex (*K* = 5) scales, which were based on patterns of male movement across multiple years of monitoring [39].

We additionally used redundancy analysis (RDA) [66] to assess the relationship between geography and genetic structure. RDA is a constrained ordination analogous to multivariate regression in which a predictor dataset (in this case, lek geography) is used to maximize the variance partitioned in a response dataset (sage-grouse genotype probabilities) [56]. RDA has been previously employed to compare the relative strengths of geography and other potential barriers to gene flow (e.g. climate, habitat) as predictors of population genetic structure (e.g. [67, 68]). We performed RDA with latitude and longitude as predictors of our genotype probability matrix using the vegan package in R. The correlation between lek latitude and longitude was−0.396 (***R***), which is smaller than the maximum correlation of RDA predictor variables (|***R***| = 0.7) suggested by [56].

### Genetic structure and habitat resistance

To further summarize genetic differentiation among leks, we calculated pairwise *F*_ST_ [69] based on allele frequencies within leks across all loci, and assessed significance using a permutation approach. As an additional metric of differentiation, we calculated Nei’s genetic distance (*D*; [70]) and used these values to generate neighbor-joining trees with the ape package [71] in R.

To assess if environmental features contribute to observed differentiation among leks, we used a landscape genetic approach that compared genetic distance (*D*) with the summed environmental resistance (a proxy for potentially reduced movement due to unsuitable habitat or barriers) between each pair of leks using a least-cost paths framework [72]. We derived a habitat resistance surface by taking the inverse of female sage-grouse nest site habitat selection model developed for this system [73] (see Fig. 1b). Additionally, we developed resistance surfaces including anthropogenic features (e.g. highways and transmission lines) as barriers at different thresholds of movement permeability. Movement barrier resistance surfaces were derived from a 100 m buffer around all roads and power lines within the study area. As outlined by [74], each pixel within 100 m of a barrier was assigned either the maximum environmental resistance value from the habitat model (permeable barriers) or assigned a resistance value four orders of magnitude greater than the maximum resistance value (low-permeable barriers). For each resistance surface, we used the landscape genetics toolbox [75] in ArcMap 10.0 (Environmental Systems Research Institute, Redlands, CA) to produce a pairwise matrix of least-cost distance paths among leks. We assessed the association between genetic distance and least-cost path for each resistance surface with Mantel tests calculated in the ecodist package [76] in R.

### The effect of genomic sampling on genetic structure estimates

To determine the number of SNPs required to capture geographic genetic structure and to examine the robustness of our results to the number of loci included in our analyses, we performed power analyses for PCA and DAPC. We constructed ten randomly sampled datasets of 22 different sizes, ranging from 50 to 27,000 SNPs (220 datasets in total). For each dataset, we conducted PCA and Procrustes analysis as described above. Additionally, we employed DAPC on each subset to determine how data set size influenced the proportions of individuals correctly assigned to lek and lek complex.

We also evaluated the possible influence of loci with exceptionally high *F*_ST_ (i.e., “outlier” loci) on genetic structure parameter estimates by generating genotype sets with highly differentiated loci removed. We generated locus-specific pairwise *F*_ST_ estimates [69] for all 45 pairwise combinations of leks and for all ten pairwise combinations of lek complexes. We removed loci with *F*_ST_ estimates above the 98th and 97th quantiles of the genome-wide *F*_ST_ distribution for any pairwise analysis at the lek and lek complex levels, and conducted PCA and Procrustes analyses on each of the four trimmed data sets.

## Results

After quality screening, we retained 169,622,388 DNA sequences from 140 individuals. The initial *de novo* assembly placed 22,175,595 reads into a set of 218,483 high-coverage contigs; the consensus sequences of these contigs were then used as a reference for the genomic regions represented by our GBS data. The final assembly contained 14 × 10^7^ reads (85 % of total reads) with an average of 838,715 reads assembled per individual. After identifying variant sites where >90 % of individuals had at least one read, we retained 27,866 SNPs with minor allele frequencies >5 % and with an average coverage of 5.7X per locus per individual (sd = 1.7). Analyses below are based on genotype probabilities estimated with the hierarchical Bayesian model of [52].

### The relationship between geography and genetic structure

**Fig. 2:**
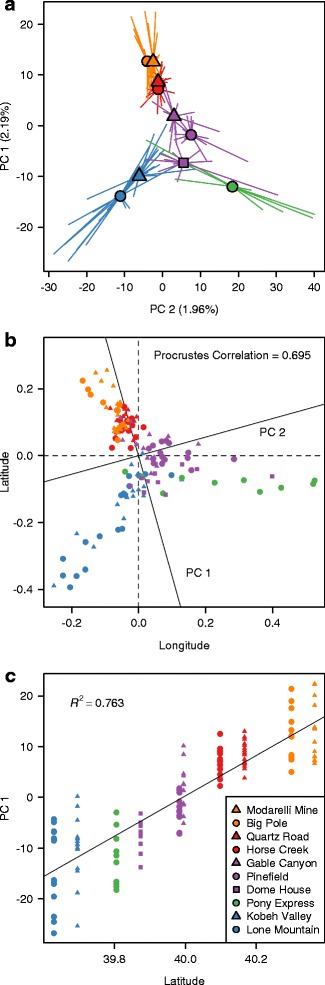
Sage-grouse population genomic structure mirrors the fine-scale geography of leks. **a** Mean PCA scores for each lek are denoted by symbols, with segments connecting means and individual points. The proportion of variation explained by each PC is listed on each axis. **b** PCA scores were rotated onto a standardized geographic matrix to maximize the Procrustes correlation (0.695) between genomic and geographic structure. **c** A regression with latitude predicting PC1. The legend is ordered from highest to lowest latitude and leks within the same lek complex share the same color (North Cortez = *orange*; Cortez = *red*; Pine Valley = *purple*; Pony Express = *Green*; Kobeh Valley = *blue*)

The first two PCs explained 2.3 and 2.0 % of the total genotypic variation (Fig. 2a) and clustered individuals from leks and lek complexes together to varying degrees. Although there was considerable overlap of adjacent leks, those separated by >30 km showed almost no overlap in PC space and lek complexes formed largely non-overlapping groups (Fig. 2a). PC1 and PC2 scores were statistically different among both individual leks (PERMANOVA, ***R***^2^ = 0.675; ***F***_9,130_ = 30.06; ***P*** < 0.001) and lek complexes (***R***^2^ = 0.644; ***F***_4,135_ = 60.97; ***P*** < 0.001). There was a hierarchical pattern of genetic structure, where lek complexes exhibited stronger and more consistent differentiation, while neighboring leks exhibited subtle if any differentiation. Additionally, the mean genetic distance among individuals sharing the same lek complex was significantly smaller than a null distribution of genetic distances (Additional file 1: Figure S2), suggesting that individuals within lek complexes have elevated relatedness (this pattern also held at the lek level; Additional file 1: Figure S2). The Procrustes correlation between genomic and geographic matrices was 0.679 (***P*** < 0.001; Fig. 2b; Additional file 1: Figure S3) and latitude explained 76.5 % of the variation in PC1 (***F***_1,138_ = 448.7; ***P*** < 0.001; Fig. 2c).

For the DAPC without *a priori* group assignments, two PCs were retained based on 20 replicate α-score tests (mean = 1.80; sd = 1.43). The *K* = 2 model fit the data best; BIC steadily increased from *K* = 2 to *K* = 40 (Additional file 1: Figure S4A). The first discriminant function (DF) clearly separated sage-grouse individuals into two clusters (Additional file 1: Figure S4B); one group mostly comprised individuals from the North Cortez and Cortez lek complexes, while the other group was composed of individuals from the Pine Valley, Pony Express, and Kobeh Valley lek complexes (Additional file 1: Figure S4C).

**Fig. 3:**
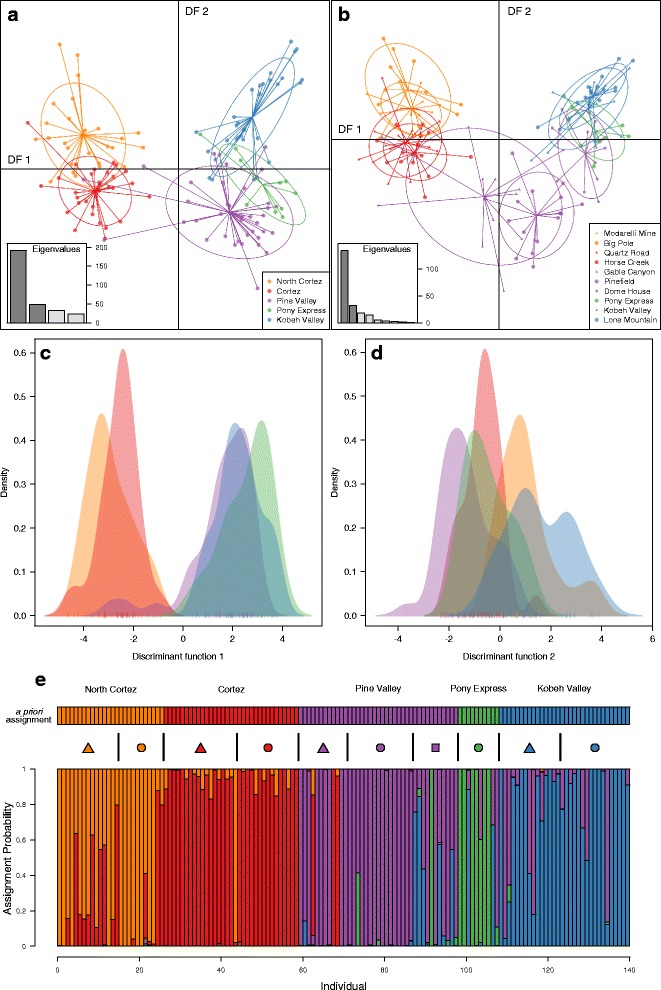
Discriminant analysis of principal components (DAPC) clusters sage-grouse individuals by *a priori* lek and lek complex designations. Scatterplots of DF1 and DF2 are displayed for both the (**a**) lek complex and (**b**) lek analyses. Insets display the eigenvalues for each DF from DAPC. For the lek complex DAPC, density distributions are displayed to aid in the visualization of how lek complexes cluster along (**c**) DF1 and (**d**) DF2. (**e**) Individual assignment probabilities for each lek complex are displayed in a bar graph, with *a priori* lek and lek complex designations displayed above. The legends are ordered from highest to lowest latitude and leks within the same lek complex share the same color (North Cortez = *orange*; Cortez = *red*; Pine Valley = *purple*; Pony Express = *Green*; Kobeh Valley = *blue*)

For the DAPC analyses using *a priori* lek assignments, 16 PCs were retained for the lek DAPC (α-score mean = 15.55; sd = 2.06) and 11 PCs were retained for the lek complex DAPC (α-score mean = 10.85; sd = 1.09). The lek complex DAPC (Fig. 3a) performed much better at clustering individuals than the lek DAPC (Fig. 3b); in both, leks within a complex largely overlapped. DF1 separated the North Cortez and Cortez lek complexes from the Pine Valley, Pony Express, and Kobeh Valley lek complexes (Fig. 3c), similar to the results from the DAPC without *a priori* group assignments (Additional file 1: Figure S4). Although DF2 largely separated the North Cortez and Cortez lek complexes, some overlap remained between the Pine Valley, Pony Express, Kobeh Valley lek complexes (Fig. 3d). Despite this, individual scores for DF1 and DF2 were significantly different for both individual leks (PERMANOVA, ***R***^2^ = 0.903; ***F***_9,130_ = 133.97; ***P*** < 0.001) and lek complexes (***R***^2^ = 0.850; ***F***_4,135_ = 191.18; ***P*** < 0.001). DAPC assigned only 48.6 % of individuals to the correct lek, but 80.0 % of individuals were assigned to the correct lek complex (Fig. 3e).

**Fig. 4:**
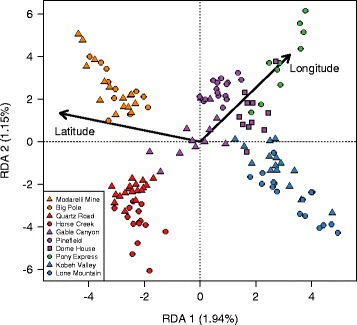
The RDA plot illustrates the strong relationship between geography and population genetic structure for sage-grouse in central Nevada. Mean RDA scores are plotted for each individual and vectors depict the direction of the two predictor variables (latitude and longitude) relative to the RDA axes. The proportion of variance explained by each RDA axis is listed on each axis label. The legend is ordered from highest to lowest latitude and leks within the same lek complex share the same color (North Cortez = *orange*; Cortez = *red*; Pine Valley = *purple*; Pony Express = *Green*; Kobeh Valley = *blue*)

The first two RDA axes (constrained by latitude and longitude) explained 1.94 and 1.15 % of the variance in total genotypic variation, respectively (Fig. 4). As in PCA and DAPC, there was a significant effect of lek (PERMANOVA, ***R***^2^ = 0.905; ***F***_9,130_ = 138.2; ***P*** < 0.001) and lek complex (***R***^2^ = 0.854; ***F***_4,135_ = 197.28; ***P*** < 0.001) designations.

### Genetic structure and habitat resistance

**Fig. 5:**
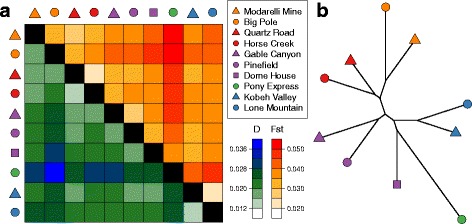
A graphical summary of genetic distances among sage-grouse leks. **a** A heat map displays pairwise genetic distance (*D*; [70]; *lower triangle*) and pairwise *F*_ST_ ([69]; *upper triangle*) among leks ordered from highest to lowest latitude. All but two pairwise *F*_ST_ estimates were significantly larger than expected (Quartz Road vs. Horse Creek and Kobeh Valley vs. Lone Mountain; see Additional file 1: Table S2 for more details). **b** A neighbor-joining tree made from *D* depicts the genetic relationships among individual leks and coincides with the results from PCA (Fig. 2) and DAPC (Fig. 3). The legend is ordered from highest to lowest latitude and leks within the same lek complex share the same color (North Cortez = *orange*; Cortez = *red*; Pine Valley = *purple*; Pony Express = *Green*; Kobeh Valley = *blue*)

**Fig. 6:**
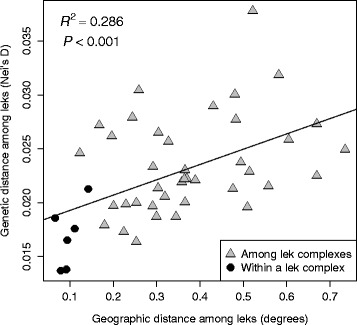
Results from a Mantel test with pairwise geographic distance among leks predicting pairwise genetic distance (*D*; [70]) are consistent with a pattern of isolation by distance (equation: y = 0.142x + 0.179)

Estimates of *D* were highly correlated with *F*_ST_ (Fig. 5a) and grouped leks within the same lek complex (with the exception of Dome House; Fig. 5b), consistent with multivariate analyses (Fig. 3). Although the pairwise *F*_ST_ estimates were quite small, all but two were significantly larger than expected (Additional file 1: Table S2). Mantel tests indicated that geographic distance most strongly predicted variation in *D* among leks (*R*^2^ = 0.286; *P* < 0.001; Table 1; Fig. 6); however, inclusion of the habitat resistance variable to the model explained an increased proportion of the variation in *D* among leks (*R*^2^ = 0.336; *P* < 0.001; Table 1)*.* We did not find any support for an influence of anthropogenic barriers on genetic distance (permeable barriers *R*^2^ = 0.080; *P* = 0.287; low permeable barriers: *R*^2^ = 0.068; *P* = 0.262; Table 1).

**Table 1:** Summary results from Mantel models testing if genetic distance (*D*; [70]) among sage-grouse leks is predicted by geographic distance, habitat suitability, and potential barriers to movement (e.g. roads and power lines)

Model variables	*R* ^2^	*P*
Distance + habitat^a^	0.336	<0.001
Distance	0.286	<0.001
Distance + permeable barriers^b^	0.080	0.287
Distance + low-permeable barriers^b^	0.068	0.262

^a^The habitat suitability resistance surface was derived from the inverse of a sage-grouse nest selection model (see Fig. 1 for more details)

^b^Movement barrier resistance surfaces were derived from a 100 m buffer around all roads and power lines within the study area. Each pixel within 100 m of a barrier was assigned either the maximum environmental resistance value observed in the habitat suitability model (Permeable Barriers) or assigned an environmental resistance value four orders of magnitude greater than the maximum environmental resistance value (Low-permeable barriers)

### The effect of genomic sampling on genetic structure estimates

**Fig. 7:**
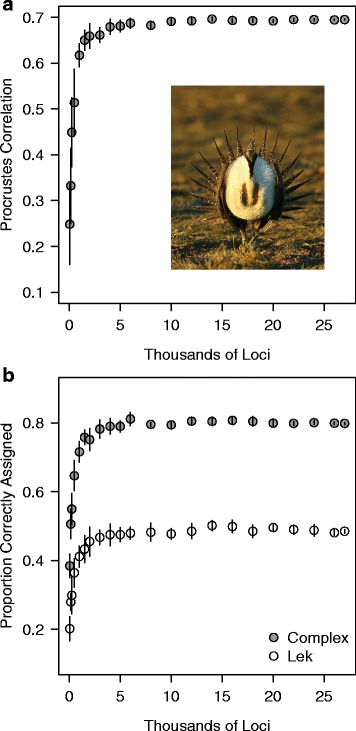
To determine the effect of increased SNP sampling on the precision and information content of population genetic analyses for this system, (**a**) Procrustes analysis and (**b**) DAPC were performed for 22 subsets of the total number of SNPs (*N* = 50, 150, 250, 500, 1 k, 2 k, 3 k, 4 k, 5 k, 6 k, 8 k, 10 k, 12 k, 14 k, 16 k, 18 k, 20 k, 22 k, 24 k, 26 k, 27 k). Ten replicates were analyzed for each subset. Note that approximately 4000 loci are needed to ensure precise estimates of genetic structure (*vertical lines* depict standard deviations for each parameter estimate). Inset depicts a male sage-grouse displaying at a lek in central Nevada (photo credit: EJB)

The magnitude and precision of Procrustes correlations between genetic and geographic matrices increased strongly in subsets containing more SNPs, ultimately plateauing around ~4000 SNPs (Fig. 7a). A similar pattern was found for DAPC, with the proportion of correctly assigned individuals reaching an asymptote at ~4000 SNPs at both the lek and lek complex scales (Fig. 7b). Thus, data sets containing fewer than ~4000 SNPs generated parameter estimates with reduced precision (i.e. larger standard deviations of parameter estimates), and failed to quantify the geographic scale of genetic structure. However, data sets containing more than ~4000 SNPs generated similar parameter estimates, indicating that the pattern of genetic structure is not driven by small numbers of exceptional loci or those residing in specific genomic regions.

We also examined how the results displayed in Fig. 2 (PCA; Procrustes; latitude vs. PC1 regression) were affected by removing loci with exceptionally high *F*_ST_ estimates. After removing loci with *F*_ST_ estimates above the 97th and 98th quantile for all pairwise lek comparisons, subsets of 22,615 and 16,630 loci were retained, while subsets of 25,991 and 23,040 loci were retained after the lek complex comparisons. Results from PCA, Procrustes, and regression analyses based on the four “outlier” trimmed genotype subsets were indistinguishable from those for the full data set (Additional file 1: Figure S5).

## Discussion

Our results indicate that lek geography and habitat features influence sage-grouse population genetic variation at a fine geographic scale across the individual, lek, and lek complex levels. Consistent with isolation by lek distance, geography explained a large proportion of individual genetic variation in ordination space (Figs. 2 and 4), predetermined delineations of lek complexes clustered together (Figs. 3 and 5), and lek-level estimates of genetic distance were strongly related to geographic distance (Table 1; Fig. 6). Although statistically significant, levels of genetic differentiation among neighboring leks were low, as indicated by the low percentage of variation explained by the first two axes of PCA, DAPC, and RDA and by the small estimates of genetic differentiation among leks (Fig. 5). Such low levels of differentiation are not surprising and are consistent with past studies based on smaller datasets not detecting genetic differentiation across geographically proximate leks (e.g. [43, 45, 47, 77]).

Sage-grouse from the different lek complexes formed distinct and statistically identifiable clusters in ordination space. The most pronounced genetic structure divided northern lek complexes (Cortez and North Cortez) from more southern complexes (Kobey Valley, Pony Express, and Pine Valley) (Figs. 2, 3 and 4, Additional file 1: Figure S4). The DAPC analysis without *a priori* information inferred *K* = 2 as the best model and assigned individuals almost entirely to these two groups (Additional file 1: Figure S4). This pattern is consistent with sage-grouse from the Cortez and North Cortez complexes predominantly sharing summer brood rearing habitat in the Cortez Range, while individuals from other complexes typically cohabitate in the Roberts Creek Range [50, 51]. Nonetheless, individuals are highly faithful to their specific leks during subsequent spring breeding seasons [39]. Additionally these results are in accord with other studies reporting that the pattern and strength of genetic structure can depend on which stage in the breeding cycle samples are collected (in this case, mating vs. brood rearing) (e.g. [15]). Within these groups, *a priori* designated lek complexes formed largely non-overlapping clusters in ordination analyses (Figs. 2, 3 and 4), with ~80 % of individuals assigned to the correct lek complex in DAPC (Fig. 3). Differentiation among neighboring leks was much less pronounced; only ~49 % of individuals were assigned to the correct lek in DAPC, and individuals from neighboring leks overlapped substantially in ordination space. Even with limited gene flow among neighboring leks, our results illustrate a hierarchical pattern of fine-scale spatial genetic structure consistent with isolation by effective lek and lek complex distance (Fig. 6).

The strong site fidelity previously documented in this system [39] could reduce gene flow among leks at some geographic scales. Capture-mark-recapture data from these leks indicate that inter-lek movements by males are very rare (~1.5 %; [39]) even though individuals have overlapping summer distributions [50, 51] and can move large distances across the landscape within a year (Additional file 1: Figure S1). By using geographic distances among leks in our population-level genetic analyses we more directly accounted for the effect of lek location on genetic structure. Nearly all of the movements we did observe were among neighboring leks, which could contribute to limited differentiation within complexes (e.g. [14]). Our landscape genetic analyses based on habitat resistance surfaces indicate that habitat suitability also influences genetic distance among leks and could reinforce the effect of site fidelity on hierarchical genetic structure at the lek and lek complex levels (Table 1). Similar to [78], we did not find evidence of an influence of anthropogenic barriers on genetic distance among leks, even though certain structures within this system (i.e. transmission lines) were associated with reduced population growth [73]. These results contrast with another study that documented significant impacts of anthropogenic barriers on population genetic structure in sage-grouse from the state of Washington [74].

Reproductive skew caused by a subset of males contributing disproportionately to reproduction [79] could also potentially influence population differentiation by reducing effective population sizes and reducing the impact of migration on genetic differentiation [22]. Empirical evidence consistent with reproductive skew and/or kin selection has been found for other bird species with lek-based or cooperative mating systems (e.g. [16, 20, 40, 80–82]); however, the strength of kin selection can be context-dependent [83]. Previous analyses of sage-grouse have not found evidence for increased relatedness within leks (e.g. [48]). Nevertheless, sage-grouse that shared lek complexes (and leks) in our study were more genetically similar than would be expected by chance (Additional file 1: Figure S2), a pattern one would predict if reproductive skew were influencing geographic genetic variation. Although it is difficult to directly account for the effects of reproductive skew and kin selection on levels of population genetic structure, our results suggest that the lek-breeding system could potentially influence the hierarchical pattern of genetic differentiation we observe. Indeed, social and reproductive behaviors are commonly associated with genetic structure in vertebrates and insects [15, 84–86]. As habitat fragmentation and reproductive skew could enhance differentiation and lead to loss of variation, such mating systems could pose special challenges for protecting genetic diversity in declining species.

Our analyses provide a finer view of geographic genetic structure than most previous studies of sage-grouse (reviewed by [87]), although some studies of lek-mating birds have detected a subtle influence of lek geography (e.g. [17, 18]). In most previous studies, genetic differentiation among sage-grouse populations has been documented when leks have been isolated by large expanses of low-quality habitat (e.g. [44, 47, 77, 88]). Studies at smaller geographic scales have typically recovered less fine-scale structure, but have found evidence for isolation by distance (e.g. [46]) and, in one case, genetic differentiation among groups of leks found in neighboring management zones [49]. It is possible that the strength of genetic structure in our study was elevated as a result of either excluding females (e.g. if females exhibit lower lek fidelity than males; [79]) or including leks that occupy a part of the range where site fidelity, relatedness, and genetic differentiation interact at different levels. However, our ability to detect such fine-scale genetic structure was likely influenced by the much larger number of loci employed in our study (but see [77]).

Not surprisingly, we found that the level of genetic structure detected and the precision of population genetic parameter estimates initially increased as the number of loci analyzed rose (Fig. 7). Larger numbers of loci (>4000) more successfully captured the relationship between genetic variation and geography (Fig. 3) and increased the fraction of individuals assigned to the correct lek and lek complex in discriminant analyses (Fig. 3). However, parameter estimates and their precision were consistent in randomly built data sets with >4000 loci (Fig. 7). In addition, parameter estimates in our analyses of the full data set of SNPs were indistinguishable from those for data sets with high *F*_ST_ outlier loci removed. This indicates that the overall patterns in our results were not strongly influenced by loci with exceptional patterns of differentiation or residing in particular genomic locations. Our results are consistent with many recent studies that illustrate how the information content of population genetic analyses can depend on the extent of genomic sampling (e.g. [89–91]). For instance, a recent study of Soay sheep found that the precision and accuracy of heritability estimates grew with increasing marker numbers (reaching an asymptote at ~18,000 SNPs), and parameter estimates including this many markers were comparable to results calculated using pedigrees [92].

## Conclusions

Along with other recent studies detecting previously unrecognized structure, our results demonstrate the promise of population genomic data sets for quantifying fine-scale, ecologically relevant population genetic variation [90, 93]. The increase in resolution such approaches could bring is highly relevant to the field of conservation biology [94] because the discovery of cryptic genetic structure in species of conservation concern has the potential to inform management strategies (e.g. [90, 95]). Indeed, the fine-scale geographic genetic structure of numerous species may be shaped in part by behavioral processes, including habitat selection (e.g. [68]), site fidelity (e.g. [15]), and reproductive skew (e.g. [17]). Future investigations utilizing genome-level data sets and covering wider ecological and geographic contexts should help to tease apart the influence of habitat features, behavioral ecology, and gene flow in shaping fine-scale genetic variation in natural populations.

## Abbreviations

BIC, bayesian information criterion; DAPC, discriminant analysis of principal components; DF, discriminant function; GBS, genotyping-by-sequencing; PC, principal component; PCA, principal component analysis; PERMANOVA, permutational multivariate analysis of variance; RDA, redundancy analysis; SNP, single nucleotide polymorphism

## Additional file

Additional file 1: Table S1. Geographic coordinates and number of individuals included in final analyses (*N* = 140) for each lek and lek complex. **Table S2**. Mean observed *F*_ST_ estimates among leks was compared to a null distribution of *F*_ST_ values (based on 100 permutations of lek assignment). **Figure S1**. Histograms display the distribution of (A) yearly and (B) summer movement distances for sage-grouse individuals in this system based on telemetry data. **Figure S2**. Histograms display the distribution of Euclidean genetic distances for 1000 permutations of randomly sampled subsets of sage grouse pairs. These permutations were used to test if sage-grouse within a (A) lek complex and (B) lek were genetically more similar than expected from a null distribution. **Figure S3**. The correlation between the genomic and geographic matrices obtained from the Procrustes analysis (0.695) is compared to the distribution of correlations from 999 random permutations. **Figure S4**. Summary results from the DAPC that did not use *a priori* group assignments are displayed. (A) Bayesian information criterion (BIC) steadily increases as the number of clusters (K) increases in the model (we selected the K value with the lowest BIC). (B) For each group, a density histogram is displayed to ease in the visualization of how individuals cluster along discriminant function one. (C) Additionally, individual assignment probabilities for each lek complex are displayed in a bar graph, with *a priori* lek and lek complex designations displayed above (results from DAPC analyses utilizing *a priori* group assignments are depicted in Fig. 3 of the main text). **Figure S5**. Results from PCA using the entire SNP dataset (panels A, D, G) were compared to subsets of data with the top 2 % (B, E, H) and top 3 % of F_ST_ (C, E, I) outliers removed. Any loci that were in the top 2 or 3 % of the F_ST_ distribution for any pairwise lek comparison were removed to create the subsets. Panels (A), (B), and (C) display scatterplots of PC1 and PC2, with symbols representing lek means and segments drawn from the means to individual scores. Panels (D), (E), and (G) depict the Procrustes correlation between the first two PCs and a standardized geographic matrix. Panels (G), (H), and (I) show the results of a regression with latitude predicting PC1. (PDF 2601 kb)
