# Supplementary material for: Fine-scale genetic structure among greater sage-grouse leks in central Nevada
Source: BMC Evol Biol. 2016 Jun 14;16:127. doi: 10.1186/s12862-016-0702-4 (PMC4908695; doi:10.1186/s12862-016-0702-4)
Supplement: Additional file 1: Table S1. — Geographic coordinates and number of individuals included in final analyses (N = 140) for each lek and lek complex. Table S2. Mean observed F ST estimates among leks was compared to a null distribution of F ST values (based on 100 permutations of lek assignment). Figure S1. Histograms display the distribution of (A) yearly and (B) summer movement distances for sage-grouse individuals in this system based on telemetry data. Figure S2. Histograms display the distribution of Euclidean genetic distances for 1000 permutations of randomly sampled subsets of sage grouse pairs. These permutations were used to test if sage-grouse within a (A) lek complex and (B) lek were genetically more similar than expected from a null distribution. Figure S3. The correlation between the genomic and geographic matrices obtained from the Procrustes analysis (0.695) is compared to the distribution of correlations from 999 random permutations. Figure S4. Summary results from the DAPC that did not use a priori group assignments are displayed. (A) Bayesian information criterion (BIC) steadily increases as the number of clusters (K) increases in the model (we selected the K value with the lowest BIC). (B) For each group, a density histogram is displayed to ease in the visualization of how individuals cluster along discriminant function one. (C) Additionally, individual assignment probabilities for each lek complex are displayed in a bar graph, with a priori lek and lek complex designations displayed above (results from DAPC analyses utilizing a priori group assignments are depicted in Fig. 3 of the main text). Figure S5. Results from PCA using the entire SNP dataset (panels A, D, G) were compared to subsets of data with the top 2 % (B, E, H) and top 3 % of FST (C, E, I) outliers removed. Any loci that were in the top 2 or 3 % of the FST distribution for any pairwise lek comparison were removed to create the subsets. Panels (A), (B), and (C) display scatterplots of PC1 and PC2, with symbols repre [file 12862_2016_702_MOESM1_ESM.pdf]

Supplementary Material

Fine-scale genetic structure among greater sage-grouse leks in central Nevada

Joshua P. Jahner, Daniel Gibson, Chava L. Weitzman, Erik J. Blomberg, James S. Sedinger, and Thomas L. Parchman

Table S1

Table S1. Geographic coordinates and number of individuals (*N*) included in final analyses (140 total) for each lek and lek complex. Leks are arranged from highest to lowest latitude.

Lek Complex	Lek	*N*	Latitude	Longitude
North Cortez	Modarelli Mine	15	40.364	-116.241
	Big Pole	11	40.299	-116.248
Cortez	Quartz Road	18	40.167	-116.399
	Horse Creek	15	40.098	-116.459
Pine Valley	Gable Canyon	12	39.995	-116.255
	Pinefield	16	39.983	-116.162
	Dome House	11	39.874	-116.180
Pony Express	Pony Express	10	39.806	-116.078
Kobeh Valley	Kobeh Valley	15	39.696	-116.205
	Lone Mountain	17	39.630	-116.247

Table S2

Table S2. Mean observed F_{ST} (Hudson et al. 1992) estimates among leks were compared to a null distribution of F_{ST} values (based on 100 permutations of lek assignment). Observed estimates residing outside the 95% confidence intervals were deemed significant (* = significantly greater; *** significantly lower).

Lek 1	Lek 2	Null Mean	Null SD	95% lower	95% upper	Observed Mean
Modarelli Mine[1]	Big Pole[1]	0.0308	0.0014	0.0305	0.0310	0.0312*
Modarelli Mine[1]	Quartz Road[2]	0.0241	0.0009	0.0240	0.0243	0.0287*
Modarelli Mine[1]	Horse Creek[2]	0.0264	0.0011	0.0261	0.0266	0.0317*
Modarelli Mine[1]	Gable Canyon[3]	0.0293	0.0013	0.0290	0.0295	0.0378*
Modarelli Mine[1]	Pinefield[3]	0.0257	0.0010	0.0255	0.0259	0.0372*
Modarelli Mine[1]	Dome House[3]	0.0303	0.0011	0.0301	0.0305	0.0406*
Modarelli Mine[1]	Pony Express[4]	0.0320	0.0012	0.0317	0.0322	0.0512*
Modarelli Mine[1]	Kobeh Valley[5]	0.0263	0.0011	0.0261	0.0265	0.0378*
Modarelli Mine[1]	Lone Mountain[5]	0.0248	0.0010	0.0246	0.0250	0.0408*
Big Pole[1]	Quartz Road[2]	0.0290	0.0012	0.0287	0.0292	0.0347*
Big Pole[1]	Horse Creek[2]	0.0309	0.0013	0.0306	0.0311	0.0387*
Big Pole[1]	Gable Canyon[3]	0.0334	0.0014	0.0332	0.0337	0.0430*
Big Pole[1]	Pinefield[3]	0.0300	0.0013	0.0297	0.0303	0.0429*
Big Pole[1]	Dome House[3]	0.0346	0.0015	0.0344	0.0349	0.0460*
Big Pole[1]	Pony Express[4]	0.0361	0.0016	0.0358	0.0364	0.0554*
Big Pole[1]	Kobeh Valley[5]	0.0307	0.0013	0.0304	0.0309	0.0419*
Big Pole[1]	Lone Mountain[5]	0.0293	0.0011	0.0291	0.0296	0.0445*
Quartz Road[2]	Horse Creek[2]	0.0244	0.0011	0.0242	0.0246	0.0245
Quartz Road[2]	Gable Canyon[3]	0.0276	0.0011	0.0274	0.0278	0.0316*
Quartz Road[2]	Pinefield[3]	0.0238	0.0009	0.0236	0.0239	0.0326*
Quartz Road[2]	Dome House[3]	0.0286	0.0012	0.0284	0.0288	0.0366*
Quartz Road[2]	Pony Express[4]	0.0302	0.0013	0.0300	0.0305	0.0481*
Quartz Road[2]	Kobeh Valley[5]	0.0243	0.0009	0.0242	0.0245	0.0343*
Quartz Road[2]	Lone Mountain[5]	0.0230	0.0010	0.0228	0.0232	0.0367*
Horse Creek[2]	Gable Canyon[3]	0.0294	0.0012	0.0292	0.0296	0.0345*
Horse Creek[2]	Pinefield[3]	0.0256	0.0009	0.0254	0.0258	0.0353*
Horse Creek[2]	Dome House[3]	0.0305	0.0011	0.0303	0.0307	0.0381*
Horse Creek[2]	Pony Express[4]	0.0319	0.0014	0.0317	0.0322	0.0493*
Horse Creek[2]	Kobeh Valley[5]	0.0263	0.0010	0.0261	0.0266	0.0363*
Horse Creek[2]	Lone Mountain[5]	0.0248	0.0010	0.0247	0.0250	0.0384*
Gable Canyon[3]	Pinefield[3]	0.0288	0.0011	0.0285	0.0290	0.0293*
Gable Canyon[3]	Dome House[3]	0.0333	0.0012	0.0331	0.0336	0.0363*
Gable Canyon[3]	Pony Express[4]	0.0348	0.0015	0.0345	0.0351	0.0480*
Gable Canyon[3]	Kobeh Valley[5]	0.0293	0.0011	0.0290	0.0295	0.0370*
Gable Canyon[3]	Lone Mountain[5]	0.0279	0.0013	0.0277	0.0282	0.0397*
Pinefield[3]	Dome House[3]	0.0299	0.0013	0.0296	0.0301	0.0312*
Pinefield[3]	Pony Express[4]	0.0314	0.0012	0.0312	0.0316	0.0443*
Pinefield[3]	Kobeh Valley[5]	0.0257	0.0009	0.0255	0.0258	0.0340*
Pinefield[3]	Lone Mountain[5]	0.0242	0.0009	0.0240	0.0244	0.0373*
Dome House[3]	Pony Express[4]	0.0361	0.0014	0.0358	0.0364	0.0396*
Dome House[3]	Kobeh Valley	0.0305	0.0012	0.0303	0.0308	0.0319*
Dome House[3]	Lone Mountain[5]	0.0290	0.0012	0.0288	0.0293	0.0352*
Pony Express[4]	Kobeh Valley[5]	0.0320	0.0011	0.0318	0.0322	0.0443*
Pony Express[4]	Lone Mountain[5]	0.0306	0.0012	0.0304	0.0309	0.0457*
Kobeh Valley[5]	Lone Mountain[5]	0.0248	0.0010	0.0246	0.0250	0.0240***

<u>Lek Complexes</u>: 1 = North Cortez; 2 = Cortez; 3 = Pine Valley; 4 = Pony Express; 5 = Kobeh Valley

Figure S1

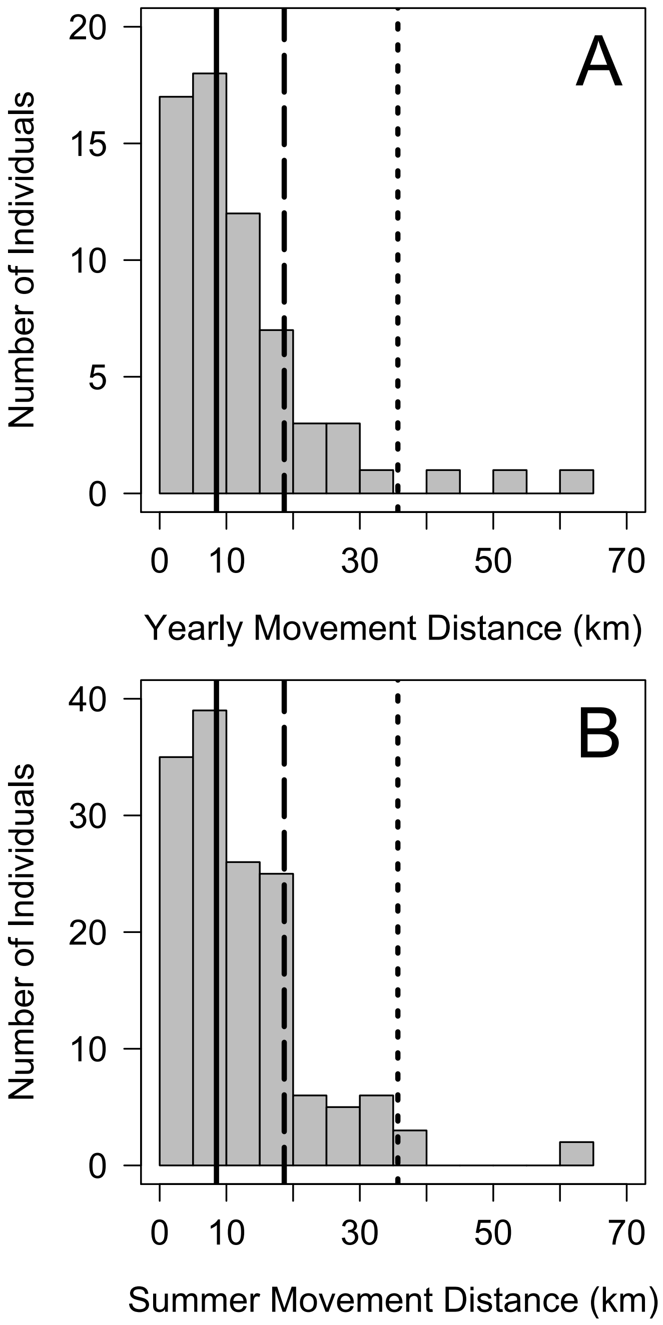

Fig. S1. Female greater sage-grouse were caught and radio-collared from 2003-2012 in Eureka County, NV. Histograms depict the (A) furthest year-round distance and (B) furthest distance in the summer (July-September; i.e. the time period associated with increased movement rates) radio-telemetered female greater sage-grouse were visually observed away from the lek of original capture. Vertical lines represent the mean distance between each study lek and the nearest neighboring lek (solid), the nearest neighboring leks outside of the lek complex (dashed), and the mean distance between all leks (dotted).

Figure S2

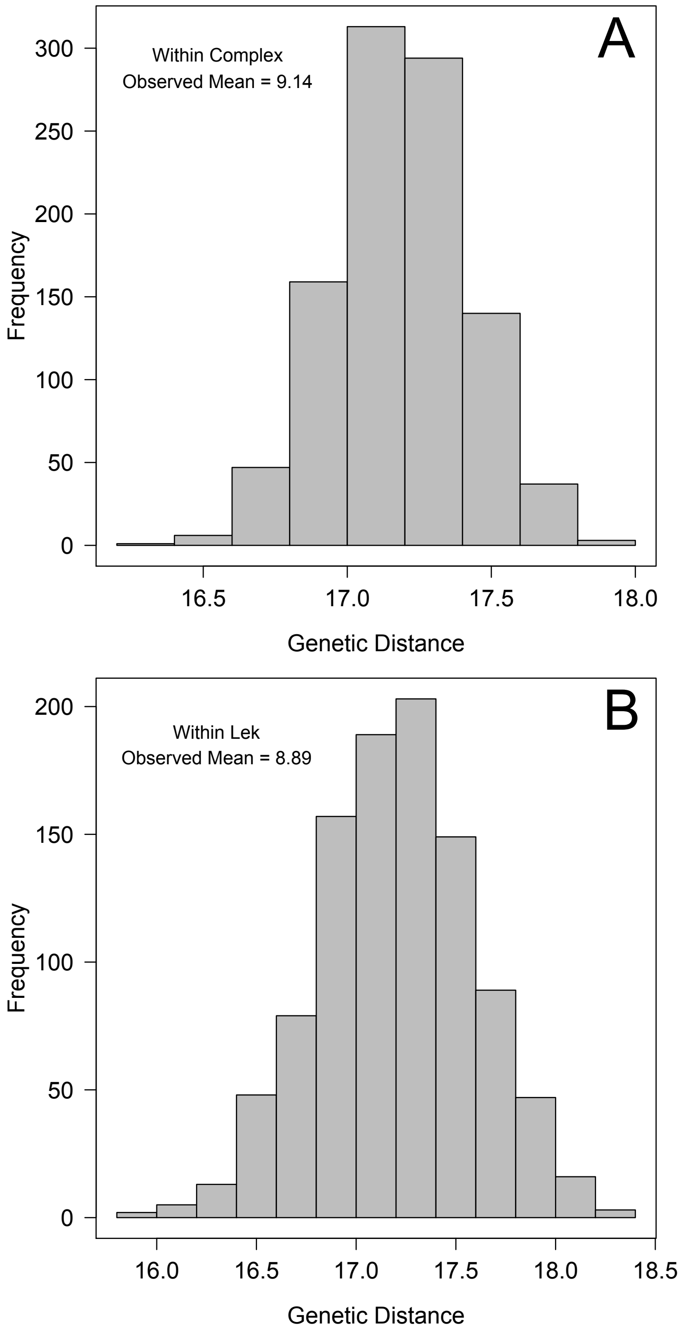

Fig. S2. To determine if sage-grouse sharing the same (A) lek complex or (B) lek have elevated relatedness, mean pairwise genetic distance among individuals residing in the same lek complex or lek was calculated as Euclidean distance of the first two PCs. Observed genetic distances were compared to null distributions of 1,000 permutations of (A) lek complex or (B) lek sharing status. In both cases, pairs who shared a lek or lek complex had significantly lower genetic distances than expected from null distributions.

Figure S3

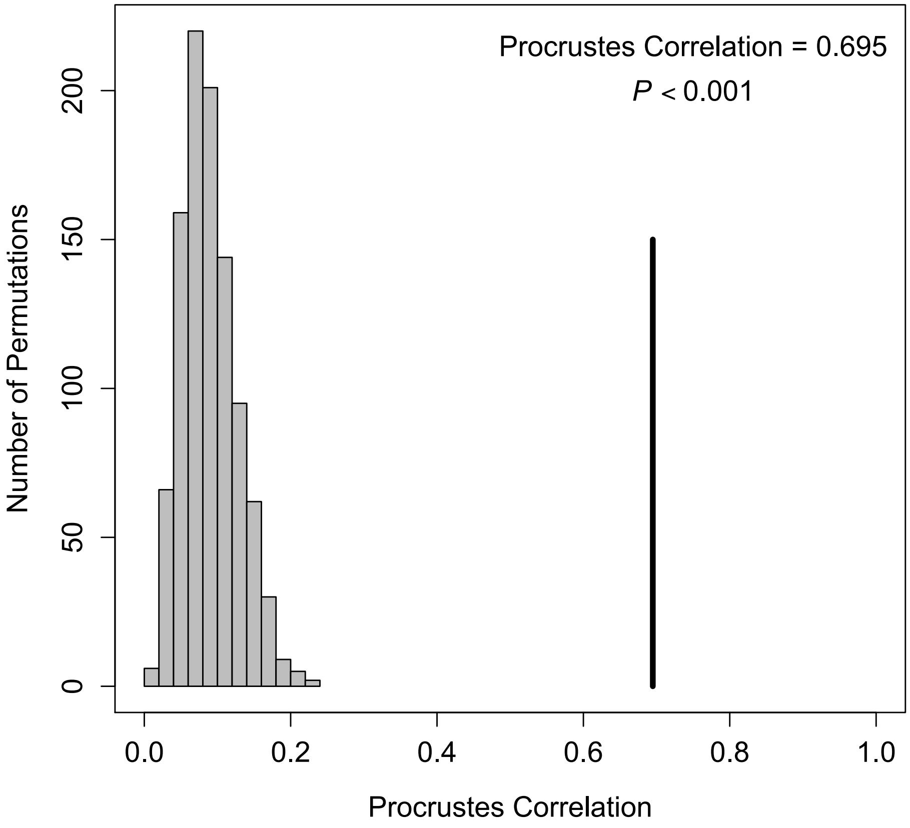

Fig. S3. The correlation between the genomic and geographic matrices obtained from the Procrustes analysis (0.695; denoted by the vertical black bar) is compared to the distribution of correlations from 999 random permutations.

Figure S4

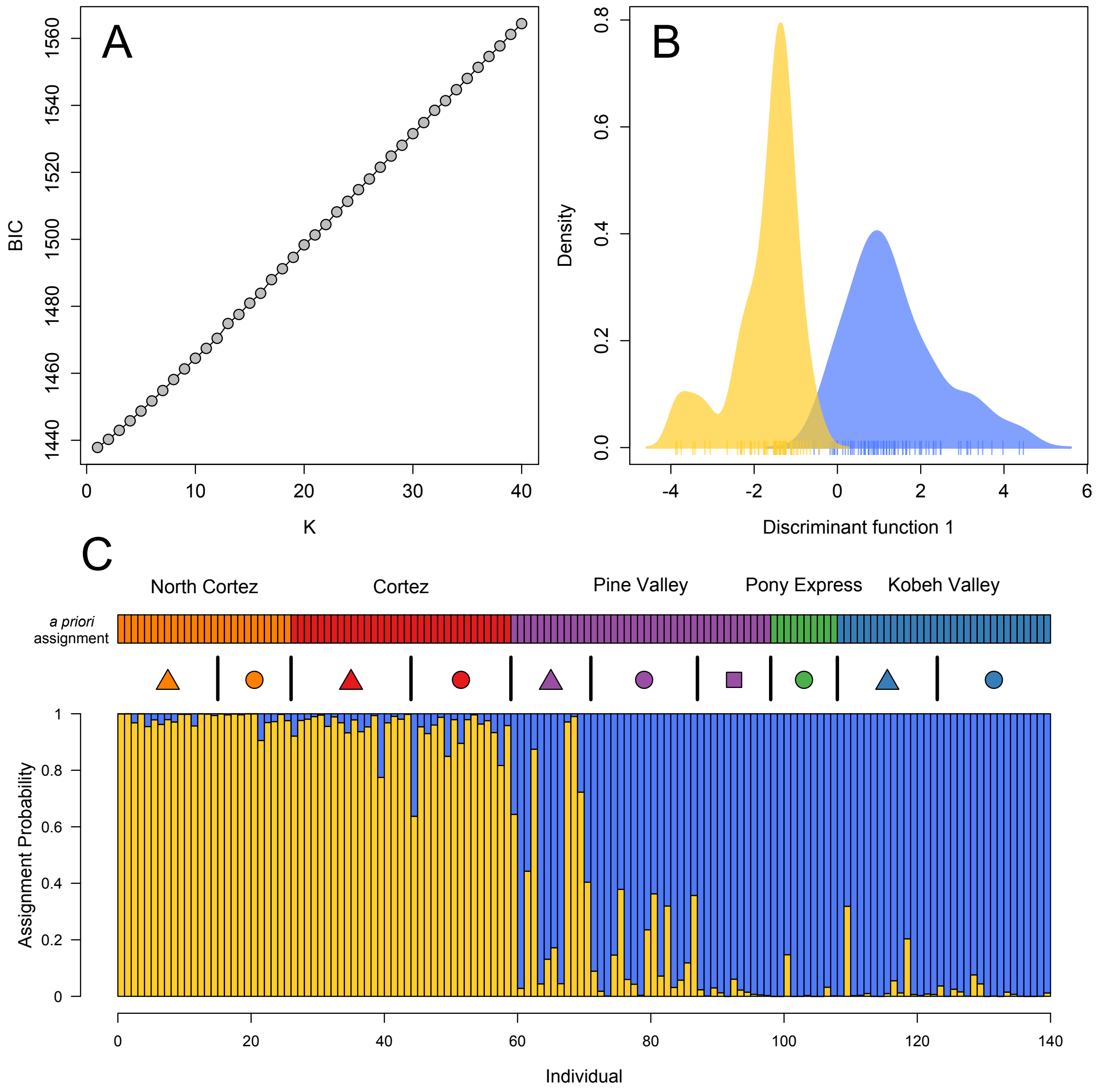

Fig. S4. Summary results from the DAPC that did not use *a priori* group assignments are displayed. (A) Bayesian information criterion (BIC) steadily increases as the number of clusters (K) increases in the model (we selected the K value with the lowest BIC). (B) For each group, a density histogram is displayed to ease in the visualization of how individuals cluster along discriminant function one. (C) Additionally, individual assignment probabilities for each lek complex are displayed in a bar graph, with *a priori* lek and lek complex designations displayed above (results from DAPC analyses utilizing *a priori* group assignments are depicted in Fig. 3 of the main text). Leks are arranged from highest to lowest latitude (left to right) and leks within the same lek complex share the same color (North Cortez = orange; Cortez = red; Pine Valley = purple; Pony Express = Green; Kobeh Valley = blue).

Figure S5

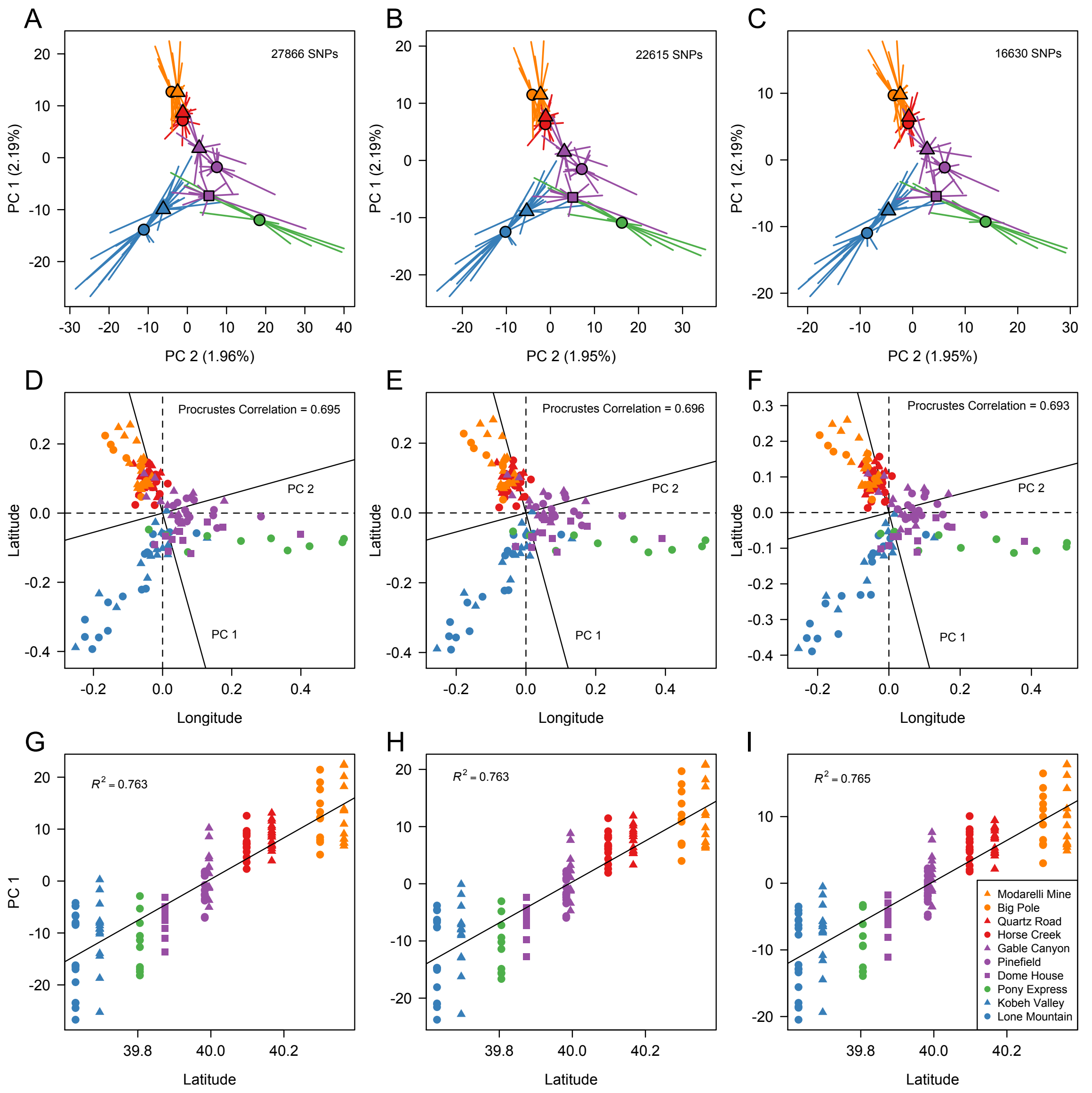

Fig. S5. Results from PCA using the entire SNP dataset (panels A, D, G) were compared to subsets of data with the top 2% (B, E, H) and top 3% of F_{ST} (C, E, I) outliers removed. Any loci that were in the top 2% or 3% of the F_{ST} distribution for any pairwise lek comparison were removed to create the subsets. Panels (A), (B), and (C) display scatterplots of PC1 and PC2, with symbols representing lek means and segments drawn from the means to individual scores. The proportion of variance explained by each PC is listed on each axis. Panels (D), (E), and (G) depict the Procrustes correlation between the first two PCs and a standardized geographic matrix. Panels (G), (H), and (I) show the results of a regression with latitude predicting PC1. The legend is ordered from highest to lowest latitude and leks within the same lek complex share the same color (North Cortez = orange; Cortez = red; Pine Valley = purple; Pony Express = Green; Kobeh Valley = blue).
